# Diagnostic Sign of Dislocation of the Thigh-Bone into the Ischiatic Notch

**Published:** 1843-10

**Authors:** 


*Diagnostic Sign of Dislocation of the Thigh-Bone into the Ischiatic Notch.*—Mr. Syme has recently narrated, in the "London and Edinburgh Monthly Journal," a case where the occurrence of the dislocation was determined by the presence of a particular sign, which appears to be of much importance in the diagnosis of a dislocation which Sir Astley Cooper has described as "the most difficult both to detect and reduce.' There is less deformity and fixture of the limb than in any other of the displacements of the thigh-bone. "This obscurity (says Mr. Syme) is much increased by attempts to effect reduction, since a moderate degree of extension almost entirely removes the shortening and inversion, which are usually considered the most characteristic symptoms. I think it, therefore, of consequence to state, that there is another feature of the injury which, according to my experience, is never absent—always well marked—and not met with in any other injury of the hip-joint, whether dislocation, fracture, or bruise. This is an arched form of the lumbar part of the spine, which cannot be straightened so long as the thigh is straight, or in a line with the patient's trunk. When the limb is raised or bent upwards upon the pelvis, the back rests flat upon the bed; but so soon as the limb is allowed to descend, the back becomes arched as before. By attention to this symptom, I have been enabled to recognise the existence of dislocation into the ischiatic notch, when it had been unnoticed by others; and, on one occasion, when it was supposed that the replacement had been effected through powerful extension by the pulleys."—*Ibid.*

